# *catena*-Poly[[bis­(*N*-ethyl­ethylene­di­amine-κ^2^ *N*,*N*′)copper(II)]-μ-cyanido-κ^2^ *N*:*C*-[dicyanido-κ^2^ *C*-palladium(II)]-μ-cyanido-κ^2^ *C*:*N*]

**DOI:** 10.1107/S160053680900885X

**Published:** 2009-03-19

**Authors:** Takashiro Akitsu, Yuki Endo

**Affiliations:** a Department of Chemistry, Faculty of Science, Tokyo University of Science, 1-3 Kagurazaka, Shinjuku-ku, Tokyo 162-8601, Japan

## Abstract

The title compound, [CuPd(CN)_4_(C_4_H_12_N_2_)_2_]_*n*_, consists of one-dimensional chains. The Cu and Pd atoms are both located on centers of symmetry in an alternating array of [Cu(N-Eten)_2_]^2+^ (N-Eten = *N*-ethyl­ethylenediamine) and [Pd(CN)_4_]^2−^ units. The Pd—C distances of 1.991 (3) and 1.992 (3) Å are inter­mediate values compared with the analogous Ni^II^ and Pt^II^ complexes [Akitsu & Einaga (2007 ▶). *Inorg. Chim. Acta*, **360**, 497–505]. Due to Jahn–Teller effects, the axial Cu—N bond distance of 2.548 (2) Å is noticeably longer than the equatorial distances [Cu—NH_2_ = 2.007 (2) and Cu—NHC_2_H_5_ = 2.050 (2) Å]. There are interchain hybrogen bonds, with N(—H)⋯N = 3.099(4) Å.

## Related literature

For photo-functional cyanide-bridged complexes, see: Escax *et al.* (2005 ▶). For Jahn–Teller switching, see: Falvello (1997 ▶). For the photo-induced and thermally accessible structural change of [Cu(en)_2_](ClO_4_)_2_ (en = ethyl­enediamine), see: Akitsu & Einaga (2003 ▶). For various coordination polymers designed so far, see: Kuchár *et al.* (2003 ▶, 2004 ▶); Petříček *et al.* (2005 ▶); Černák *et al.* (1998 ▶); Černák & Abboud (2002 ▶); Manna *et al.* (2007 ▶). Ni(en)_2_ *M*(CN)_4_ affords slightly elongated or compressed octa­hedral coordination geometries for *M* = Ni^II^ or Pd^II^, see: Černák *et al.* (1988 ▶). For related complexes, see: [Cu(en)_2_][Ni(CN)_4_] (Lokaj *et al.*, 1991 ▶); [Cu(en)_2_][Pd(CN)_4_] (Černák *et al.*, 2001 ▶); [Cu(en)_2_][Pt(CN)_4_] (Akitsu & Einaga, 2006*a* ▶). For isotypic structures, see: [Cu(N-Eten)_2_][Ni(CN)_4_] and [Cu(N-Eten)_2_][Pt(CN)_4_] (Akitsu & Einaga, 2007 ▶). For a related mononuclear complex, see: Grenthe *et al.* (1979 ▶). For the two-dimensional Cu^II^–Co^III^(CN)_6_ complex, see: Akitsu & Einaga (2006*b* ▶). For tetra­gonal Jahn–Teller distortion, see: Hathaway & Billing (1970 ▶). For a mononuclear Cu^II^ complex without Jahn–Teller distortion, see: Zibaseresht & Hartshorn (2006 ▶).
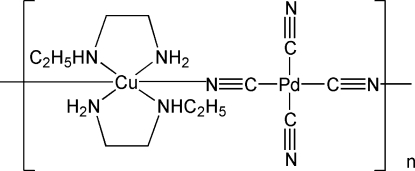


## Experimental

### 

#### Crystal data

[CuPd(CN)_4_(C_4_H_12_N_2_)_2_]
*M* *_r_* = 450.33
Triclinic, 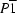

*a* = 7.360 (4) Å
*b* = 7.567 (4) Å
*c* = 9.061 (4) Å
α = 69.091 (5)°
β = 72.490 (6)°
γ = 89.680 (6)°
*V* = 446.6 (4) Å^3^
*Z* = 1
Mo *K*α radiation
μ = 2.21 mm^−1^
*T* = 296 K
0.20 × 0.15 × 0.10 mm

#### Data collection

Brruker SMART CCD area-detector diffractometer
Absorption correction: multi-scan (*SADABS*; Bruker, 1998 ▶) *T*_min_ = 0.662, *T*_max_ = 0.806
2943 measured reflections
1934 independent reflections
1763 reflections with *I* > 2σ(*I*)
*R*_int_ = 0.027

#### Refinement

*R*[*F* ^2^ > 2σ(*F* ^2^)] = 0.034
*wR*(*F* ^2^) = 0.105
*S* = 0.85
1934 reflections
105 parameters
H-atom parameters constrained
Δρ_max_ = 1.24 e Å^−3^
Δρ_min_ = −1.28 e Å^−3^

### 

Data collection: *SMART* (Bruker, 1998 ▶); cell refinement: *SAINT* (Bruker, 1998 ▶); data reduction: *SAINT*; program(s) used to solve structure: *SIR92* (Altomare *et al.*, 1994 ▶); program(s) used to refine structure: *SHELXL97* (Sheldrick, 2008 ▶); molecular graphics: *ORTEPII* (Johnson, 1976 ▶); software used to prepare material for publication: *SHELXL97*.

## Supplementary Material

Crystal structure: contains datablocks global, I. DOI: 10.1107/S160053680900885X/bg2245sup1.cif

Structure factors: contains datablocks I. DOI: 10.1107/S160053680900885X/bg2245Isup2.hkl

Additional supplementary materials:  crystallographic information; 3D view; checkCIF report

## Figures and Tables

**Table 1:** Hydrogen-bond geometry (Å, °)

*D*—H⋯*A*	*D*—H	H⋯*A*	*D*⋯*A*	*D*—H⋯*A*
N3—H3*C*⋯N2^i^	0.90	2.26	3.099 (4)	156

Symmetry code: (i) 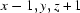.

**Fig. 1.**
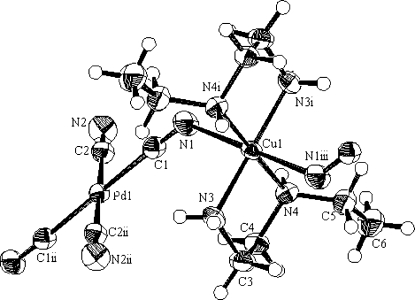
The molecular structure of (I), showing the atom-labelling scheme. Displacement ellipsoids are drawn at the 50% probability level. Symmetry codes: (i) -x, -y, 2 - z, (ii) 1 - x, 1 - y, 1 - z, (iii) x - 1, y - 1, z + 1.

## supplementary crystallographic information

### Comment

Associated with certain photo-functional cyanide-bridged complexes, Escax *et al.* (2005) have focused on the importance that structural strain of the lattice weaken ligand field strength of cyanide ligands. Additionally, so called Jahn-Teller switching (Falvello, 1997) may be a new mechanism for structural and electronic states switching even for cyanide-bridged coordination polymers containing a Cu^II^ moiety. We have reported photo-induced and thermally accessible structural change of [Cu(en)_2_](ClO_4_)_2_ (en = ethylenediamine; Akitsu & Einaga, 2003). Moreover, numerous coordination polymers, such as one-dimensional Cu^II^—Ni(CN)_4_ (Kuchár *et al.*, 2003), Cd^II^—Ni(CN)_4_ (Petříček *et al.*, 2005), Cu^II^—Pd(CN)_4_ (Kuchár *et al.*, 2004), Cu^II^—Ag_2_(CN)_3_ (Černák *et al.*, 1998), two-dimensional Cu^I^/Cu^II^—Ni(CN)_4_ (Černák *et al.*, 2002), and *cis* and *trans* Cu^II^—Pd(CN)_4_ complexes (Manna *et al.*, 2007) have been designed so far. Among them, it has been reported  that Ni(en)_2_M(CN)_4_ affords slightly elongated or compressed octahedral coordination geometries for *M* = Ni^II^ or Pd^II^, respectively (Černák *et al.*, 1988). In this context, we are interested in isostructral complexes by element-substitution and their structural differences, for example, [Cu(en)_2_][Ni(CN)_4_] (Lokaj *et al.*, 1991), [Cu(en)_2_][Pd(CN)_4_] (Černák *et al.*, 2001), and [Cu(en)_2_][Pt(CN)_4_] (Akitsu & Einaga, 2006*a*). Because we have already reported [Cu(N-Eten)_2_][Ni(CN)_4_] and [Cu(N-Eten)_2_][Pt(CN)_4_] complexes (Akitsu & Einaga, 2007), we report herein [Cu(N-Eten)_2_][Pd(CN)_4_](I)in order to investigate stereochemical effects by ethyl groups as the second series.

Compound (I) consists of one-dimensional chains (Fig. 1). Both Cu and Pd atoms are located on centers of symmetry in the alternative array of [Cu(N-Eten)_2_]^2+^ and [Pd(CN)_4_]^2-^ moieties(Fig. 2). The Pd—C bond distances of (I) (Table 1) and the unit cell volume of (I) (446.6 (4) Å^3^) is middle value among the corresponding Ni^II^ (438.5 (5) Å^3^) and Pt^II^ (448.5 (3) Å^3^) complexes (Akitsu & Einaga, 2007). As for the [Cu(en)_2_][*M*(CN)_4_] series, similar features were also observed in Ni^II^ (333.9 (9) Å^3^) (Lokaj *et al.*, 1991), Pd^II^ (347.63 (6) Å^3^) (Černák *et al.*, 2001), and Pt^II^ (353.9 (4) Å^3^) (Akitsu & Einaga, 2006*a*), which are mainly attributed to gradual changes of ionic radii of Ni^II^, Pd^II^, and Pt^II^ ions.

The geometry of the [Cu(N-Eten)_2_]^2+^ unit in (I) is similar to the related mononuclear (Grenthe *et al.*, 1979) and two-dimensional Cu^II^—Co^III^(CN)_6_ (Akitsu & Einaga, 2006*b*) complexes.

Due to Jahn–Teller effects the axial Cu—N bond distance of 2.548 (2) Å is sensibly longer than the equatorial ones, (NH_2_) 2.007 (2) and (NHC_2_H_5_) 2.050 (2) Å. However, it should be noted that ethyl groups gave characteristic strain to the crystal lattice and deviate from clearly gradual structural changes of the [Cu(N-Eten)_2_][*M*(CN)_4_] series. The axial Cu1—N1 bond length of 2.548 (2) Å in (I) is comparable to the analogous Ni^II^ (2.554 (2) Å) and Pt^II^ (2.550 (3) Å) complexes (Akitsu & Einaga, 2007). The degree of tetragonal Jahn–Teller distortion of [Cu(N-Eten)_2_]^2+^ moiety in (I) is T = 0.796 (mean T is the ratio of in-plane Cu—N bond lengths / axial Cu—N bond lengths; Hathaway & Billing, 1970). The T values are 0.796 and 0.797 for the analogous Ni^II^ and Pt^II^ complexes, respectively. On the other hand, as for [Cu(en)_2_][*M*(CN)_4_] series, the axial Cu—N bond lengths exhibited gradual changes for Ni^II^(2.533 (4) Å, Lokaj *et al.*, 1991), Pd^II^ (2.544 (2) Å, Černák *et al.*, 2001), and Pt^II^ (2.562 (5) Å, Akitsu & Einaga, 2006*a*) complexes, respectively. Interestingly, absence of Jahn-Teller distortion is also reported for a certain mononuclear Cu^II^ complex (Zibaseresht & Hartshorn, 2006). In (I), there are N—H^···^N hydrogen bonds (Table 2), though some H^···^N distances are longer than the common values.

### Experimental

The compound (I) was obtained by slow diffusion of a methanol solution (36 ml) of [Cu(N-Eten)_2_](NO_3_)_2_ (36.0 mg, 0.100 mmol) onto an aqueous solution (5 ml) of K_2_[Pd(CN)_4_] (29.0 mg, 0.100 mmol) at 298 K. After several days, blue single crystals of (I) were obtained from the surface (Yield: 34.4 mg, 76.6%). Anal. Calcd for C_12_H_24_CuN_8_Pd: C 32.00, H 5.37, N 24.88%. Found: C 32.08, H 5.13, N 25.00%. IR (KBr, ν, cm^-1^): 470, 665, 721, 981, 1068, 1096, 1156, 1377, 1464, 1591, 2129 and 2132 (cyanide), 2853, 2923, 2953, 3162, 3253, 3273, 3310, 3582. Electronic spectrum (diffuse reflectance): 18100 cm^-1^ (F(*R*_d_) 1.73) (d-d transition of distorted octahedral Cu^II^ ion). Weiss constant = -7.76 K (antiferromagnteic interaction). XPS Cu 2p_1/2_ 960, Cu Cu 2p_3/2_ 940 eV (Cu^II^), Pd 3d_3/2_ 357, and Pd 3d_5/2_ 352 eV (Pd^II^).

### Refinement

H atoms bonded to C and N atoms were placed in calculated positions, with C—H = 0.97 or 0.96 Å and N—H = 0.91 or 0.90 Å and with *U*_iso_(H) = 1.2*U*_eq_(C and N), and included in the final cycles of refinement using riding constraints.

### Crystal data

**Table:**

[CuPd(CN)_4_(C_4_H_12_N_2_)_2_]	*Z* = 1
*M**_r_* = 450.33	*F*(000) = 227
Triclinic, *P*1	*D*_x_ = 1.674 Mg m^−^^3^
Hall symbol: -P 1	Mo *K*α radiation, λ = 0.71073 Å
*a* = 7.360 (4) Å	Cell parameters from 1805 reflections
*b* = 7.567 (4) Å	θ = 2.5–27.5°
*c* = 9.061 (4) Å	µ = 2.21 mm^−^^1^
α = 69.091 (5)°	*T* = 296 K
β = 72.490 (6)°	Prismatic, blue violet
γ = 89.680 (6)°	0.20 × 0.15 × 0.10 mm
*V* = 446.6 (4) Å^3^	

### Data collection

**Table:**

Brruker SMART CCD area-detector diffractometer	1934 independent reflections
Radiation source: fine-focus sealed tube	1763 reflections with *I* > 2σ(*I*)
graphite	*R*_int_ = 0.027
φ and ω scans	θ_max_ = 27.5°, θ_min_ = 2.5°
Absorption correction: multi-scan (*SADABS*; Bruker, 1998)	*h* = −8→9
*T*_min_ = 0.662, *T*_max_ = 0.806	*k* = −4→9
2943 measured reflections	*l* = −7→11

### Refinement

**Table:**

Refinement on *F*^2^	Primary atom site location: structure-invariant direct methods
Least-squares matrix: full	Secondary atom site location: difference Fourier map
*R*[*F*^2^ > 2σ(*F*^2^)] = 0.034	Hydrogen site location: mixed
*wR*(*F*^2^) = 0.105	H-atom parameters constrained
*S* = 0.85	*w* = 1/[σ^2^(*F*_o_^2^) + (0.1*P*)^2^] where *P* = (*F*_o_^2^ + 2*F*_c_^2^)/3
1934 reflections	(Δ/σ)_max_ < 0.001
105 parameters	Δρ_max_ = 1.24 e Å^−^^3^
0 restraints	Δρ_min_ = −1.28 e Å^−^^3^

### Special details

**Table:**

Experimental. Refinement of F^2^ against ALL reflections. The weighted *R*-factor *wR* and goodness of fit S are based on F^2^, conventional *R*-factors *R* are based on F, with F set to zero for negative F^2^. The threshold expression of F^2^ > 2sigma(F^2^) is used only for calculating *R*-factors(gt) *etc*. and is not relevant to the choice of reflections for refinement. *R*-factors based on F^2^ are statistically about twice as large as those based on F, and R– factors based on ALL data will be even larger.
Geometry. All e.s.d.'s (except the e.s.d. in the dihedral angle between two l.s. planes) are estimated using the full covariance matrix. The cell e.s.d.'s are taken into account individually in the estimation of e.s.d.'s in distances, angles and torsion angles; correlations between e.s.d.'s in cell parameters are only used when they are defined by crystal symmetry. An approximate (isotropic) treatment of cell e.s.d.'s is used for estimating e.s.d.'s involving l.s. planes.

### Fractional atomic coordinates and isotropic or equivalent isotropic displacement parameters (Å^2^)

**Table:**

	*x*	*y*	*z*	*U*_iso_*/*U*_eq_	
Pd1	0.5000	0.5000	0.5000	0.02494 (14)	
Cu1	0.0000	0.0000	1.0000	0.02702 (16)	
N1	0.3378 (3)	0.1070 (3)	0.7927 (3)	0.0451 (6)	
N2	0.8047 (4)	0.3301 (4)	0.2789 (3)	0.0489 (6)	
N3	−0.0787 (3)	0.2641 (3)	0.9459 (3)	0.0325 (5)	
H3C	−0.1484	0.2798	1.0398	0.039*	
H3D	0.0256	0.3510	0.8958	0.039*	
N4	−0.0720 (3)	0.0022 (3)	0.7978 (2)	0.0302 (4)	
H4C	0.0309	−0.0288	0.7299	0.036*	
C1	0.4025 (3)	0.2473 (4)	0.6839 (3)	0.0323 (5)	
C2	0.6910 (4)	0.3871 (3)	0.3619 (3)	0.0318 (5)	
C3	−0.1934 (4)	0.2879 (4)	0.8339 (3)	0.0414 (6)	
H3A	−0.2025	0.4219	0.7785	0.050*	
H3B	−0.3220	0.2234	0.8969	0.050*	
C4	−0.0960 (4)	0.2037 (4)	0.7076 (3)	0.0381 (6)	
H4A	−0.1728	0.2089	0.6362	0.046*	
H4B	0.0282	0.2754	0.6384	0.046*	
C5	−0.2390 (4)	−0.1323 (4)	0.8340 (3)	0.0398 (6)	
H5A	−0.3545	−0.0876	0.8880	0.048*	
H5B	−0.2271	−0.2555	0.9118	0.048*	
C6	−0.2605 (5)	−0.1566 (5)	0.6817 (5)	0.0580 (9)	
H6A	−0.3726	−0.2434	0.7139	0.070*	
H6B	−0.1493	−0.2063	0.6301	0.070*	
H6C	−0.2732	−0.0355	0.6041	0.070*	

### Atomic displacement parameters (Å^2^)

**Table:**

	*U*^11^	*U*^22^	*U*^33^	*U*^12^	*U*^13^	*U*^23^
Pd1	0.02339 (19)	0.0266 (2)	0.01896 (19)	0.00152 (13)	−0.00348 (13)	−0.00440 (13)
Cu1	0.0346 (3)	0.0233 (3)	0.0227 (3)	0.0049 (2)	−0.0126 (2)	−0.0053 (2)
N1	0.0370 (12)	0.0373 (13)	0.0405 (13)	−0.0009 (10)	−0.0029 (10)	0.0013 (10)
N2	0.0459 (14)	0.0506 (14)	0.0457 (14)	0.0090 (12)	−0.0039 (12)	−0.0219 (12)
N3	0.0380 (12)	0.0271 (10)	0.0255 (10)	0.0025 (9)	−0.0059 (9)	−0.0052 (8)
N4	0.0275 (10)	0.0363 (11)	0.0248 (10)	0.0044 (8)	−0.0080 (8)	−0.0096 (8)
C1	0.0256 (11)	0.0370 (13)	0.0289 (12)	0.0042 (10)	−0.0041 (9)	−0.0099 (10)
C2	0.0312 (12)	0.0311 (12)	0.0270 (12)	0.0027 (10)	−0.0049 (10)	−0.0074 (9)
C3	0.0406 (15)	0.0352 (13)	0.0427 (16)	0.0100 (12)	−0.0156 (12)	−0.0061 (11)
C4	0.0445 (15)	0.0374 (13)	0.0264 (12)	0.0022 (12)	−0.0167 (11)	−0.0004 (10)
C5	0.0377 (14)	0.0421 (15)	0.0399 (14)	0.0006 (12)	−0.0134 (12)	−0.0146 (12)
C6	0.066 (2)	0.058 (2)	0.074 (2)	0.0132 (17)	−0.0404 (19)	−0.0370 (18)

### Geometric parameters (Å, °)

**Table:**

Pd1—C2	1.991 (3)	N4—C5	1.479 (3)
Pd1—C2^i^	1.991 (3)	N4—C4	1.489 (3)
Pd1—C1^i^	1.992 (3)	N4—H4C	0.9100
Pd1—C1	1.992 (3)	C3—C4	1.500 (4)
Cu1—N1	2.548 (2)	C3—H3A	0.9700
Cu1—N3^ii^	2.007 (2)	C3—H3B	0.9700
Cu1—N3	2.007 (2)	C4—H4A	0.9700
Cu1—N4^ii^	2.050 (2)	C4—H4B	0.9700
Cu1—N4	2.050 (2)	C5—C6	1.508 (4)
N1—C1	1.141 (3)	C5—H5A	0.9700
N2—C2	1.140 (3)	C5—H5B	0.9700
N3—C3	1.470 (3)	C6—H6A	0.9600
N3—H3C	0.9000	C6—H6B	0.9600
N3—H3D	0.9000	C6—H6C	0.9600
			
C2—Pd1—C2^i^	180.000 (1)	N2—C2—Pd1	177.0 (2)
C2—Pd1—C1^i^	87.83 (10)	N3—C3—C4	107.8 (2)
C2^i^—Pd1—C1^i^	92.17 (10)	N3—C3—H3A	110.1
C2—Pd1—C1	92.17 (10)	C4—C3—H3A	110.1
C2^i^—Pd1—C1	87.83 (10)	N3—C3—H3B	110.1
C1^i^—Pd1—C1	179.999 (1)	C4—C3—H3B	110.1
N3^ii^—Cu1—N3	180.0	H3A—C3—H3B	108.5
N3^ii^—Cu1—N4^ii^	85.55 (9)	N4—C4—C3	108.5 (2)
N3—Cu1—N4^ii^	94.45 (9)	N4—C4—H4A	110.0
N3^ii^—Cu1—N4	94.45 (9)	C3—C4—H4A	110.0
N3—Cu1—N4	85.55 (9)	N4—C4—H4B	110.0
N4^ii^—Cu1—N4	180.0	C3—C4—H4B	110.0
C3—N3—Cu1	107.38 (16)	H4A—C4—H4B	108.4
C3—N3—H3C	110.2	N4—C5—C6	113.9 (2)
Cu1—N3—H3C	110.2	N4—C5—H5A	108.8
C3—N3—H3D	110.2	C6—C5—H5A	108.8
Cu1—N3—H3D	110.2	N4—C5—H5B	108.8
H3C—N3—H3D	108.5	C6—C5—H5B	108.8
C5—N4—C4	112.8 (2)	H5A—C5—H5B	107.7
C5—N4—Cu1	116.00 (15)	C5—C6—H6A	109.5
C4—N4—Cu1	105.94 (16)	C5—C6—H6B	109.5
C5—N4—H4C	107.2	H6A—C6—H6B	109.5
C4—N4—H4C	107.2	C5—C6—H6C	109.5
Cu1—N4—H4C	107.2	H6A—C6—H6C	109.5
N1—C1—Pd1	176.2 (2)	H6B—C6—H6C	109.5
			
N4^ii^—Cu1—N3—C3	−163.23 (16)	Cu1—N3—C3—C4	−42.9 (2)
N4—Cu1—N3—C3	16.78 (16)	C5—N4—C4—C3	88.7 (3)
N3^ii^—Cu1—N4—C5	66.55 (19)	Cu1—N4—C4—C3	−39.2 (2)
N3—Cu1—N4—C5	−113.45 (19)	N3—C3—C4—N4	55.7 (3)
N3^ii^—Cu1—N4—C4	−167.52 (16)	C4—N4—C5—C6	70.2 (3)
N3—Cu1—N4—C4	12.47 (16)	Cu1—N4—C5—C6	−167.4 (2)

Symmetry codes: (i) −*x*+1, −*y*+1, −*z*+1; (ii) −*x*, −*y*, −*z*+2.

### Hydrogen-bond geometry (Å, °)

**Table:**

*D*—H···*A*	*D*—H	H···*A*	*D*···*A*	*D*—H···*A*
N3—H3C···N2^iii^	0.90	2.26	3.099 (4)	156

Symmetry codes: (iii) *x*−1, *y*, *z*+1.

## References

[bb1] Akitsu, T. & Einaga, Y. (2003). *Bull. Chem. Soc. Jpn*, **77**, 763–764.

[bb2] Akitsu, T. & Einaga, Y. (2006*a*). *Acta Cryst.* E**62**, m862–m864.

[bb3] Akitsu, T. & Einaga, Y. (2006*b*). *Acta Cryst.* E**62**, m750–m752.

[bb4] Akitsu, T. & Einaga, Y. (2007). *Inorg. Chim. Acta*, **360**, 497–505.

[bb5] Altomare, A., Cascarano, G., Giacovazzo, C., Guagliardi, A., Burla, M. C., Polidori, G. & Camalli, M. (1994). *J. Appl. Cryst.* **27**, 435.

[bb6] Bruker (1998). *SMART*, *SAINT* and *SADABS* Bruker AXS Inc., Madison, Wisconsin, USA.

[bb7] Černák, J. & Abboud, K. A. (2002). *Acta Cryst.* C**58**, m167–m170. 10.1107/s0108270101021898 11870291

[bb8] Černák, J., Chomič, J., Baloghová, D. & Dunaj-Jurčo, M. (1988). *Acta Cryst.* C**44**, 1902–1905.

[bb9] Černák, J., Chomič, J., Gravereau, P., Orendacova, A., Orendac, M., Kovac, J., Feher, A. & Kappenstein, C. (1998). *Inorg. Chim. Acta*, **281**, 134–140.

[bb10] Černák, J., Skorsepa, J., Abboud, K. A., Meisel, M. W., Orendac, M., Orendacova, A. & Feher, A. (2001). *Inorg. Chim. Acta*, **326**, 3–8.

[bb11] Escax, V., Champion, G., Arrio, M.-A., Zacchigna, M., Cartier dit Moulin, C. & Bleuzen, A. (2005). *Angew. Chem. Int. Ed.* **44**, 4798–4801. 10.1002/anie.200500647 15988774

[bb12] Falvello, L. R. (1997). *J. Chem. Soc. Dalton Trans.* pp. 4463–4475.

[bb13] Grenthe, I., Paoletti, P., Sandstorm, M. & Glikberg, S. (1979). *Inorg. Chem.* **18**, 2687–2692.

[bb14] Hathaway, B. J. & Billing, D. E. (1970). *Coord. Chem. Rev.* **5**, 143–207.

[bb15] Johnson, C. K. (1976). *ORTEPII* Report ORNL-5138. Oak Ridge National Laboratory, Tennessee, USA.

[bb16] Kuchár, J., Černák, J. & Abboud, K. A. (2004). *Acta Cryst.* C**60**, m492–m494. 10.1107/S0108270104019146 15467118

[bb17] Kuchár, J., Černák, J., Mayerova, Z., Kubacek, P. & Zak, Z. (2003). *Solid State Phenom* **90–91**, 323–328.

[bb18] Lokaj, J., Gyerová, K., Sopková, A., Sivý, J., Kettmann, V. & Vrábel, V. (1991). *Acta Cryst.* C**47**, 2447–2448.

[bb19] Manna, S. C., Ribas, J., Zangrando, E. & Chaudhuri, N. R. (2007). *Polyhedron*, **26**, 3189–3198.

[bb20] Petříček, V., Dušek, M. & Černák, J. (2005). *Acta Cryst.* B**61**, 280–286. 10.1107/S0108768105012632 15914892

[bb21] Sheldrick, G. M. (2008). *Acta Cryst.* A**64**, 112–122. 10.1107/S0108767307043930 18156677

[bb22] Zibaseresht, R. & Hartshorn, R. M. (2006). *Acta Cryst.* E**62**, i19–i22.

